# Supplementary material for: Fabrication of cell culture hydrogels by robotic liquid handling automation for high-throughput drug testing
Source: Commun Eng. 2025 Dec 22;4:222. doi: 10.1038/s44172-025-00575-3 (PMC12749810; doi:10.1038/s44172-025-00575-3)
Supplement: Supplementary file 2 — Supplementary Information [file 44172_2025_575_MOESM2_ESM.pdf]

Supplementary Information for

Fabrication of cell culture hydrogels by robotic liquid handling automation for high-throughput drug testing

Eloisa Torchia[1], Moises Di Sante[1], Bohdana Horda[1], Marko Mihajlovic[2], Julius Zimmermann[1], Melissa Pezzotti[1], Elisa Cimetta[3], Sylvain Gabriele[4], Ferdinando Auricchio[5], Johan Ulrik Lind[2], Alessandro Enrico[1,*], Francesco Silvio Pasqualini[1,*].

1. E. Torchia, M. Di Sante, B. Horda, J. Zimmermann, M. Pezzotti, A. Enrico, F. S. Pasqualini
   Synthetic Physiology Lab, Department of Civil Engineering and Architecture, University of Pavia, 27100, Pavia, Italy

2. M. Mihajlovic, J. U. Lind
   Department of Health Technology, Technical University of Denmark, 2800 Kgs. Lyngby, Denmark

3. E. Cimetta
   Department of Industrial Engineering, University of Padua, 35131 Padova, Italy

4. S. Gabriele
   Mechanobiology & Biomaterials Group, Research Institute for Biosciences, University of Mons, CIRMAP, 20 Place du Parc, Mons B-7000, Belgium

5. F. Auricchio
   Group of Computational Mechanics and Advanced Materials, Department of Civil Engineering and Architecture, University of Pavia, 27100, Pavia, Italy

*Corresponding authors: Email: francesco.pasqualini@unipv.it, alessandro.enrico@unipv.it

This file includes:

- Fig. S1 to S13
- Supplementary Table S1 and S2

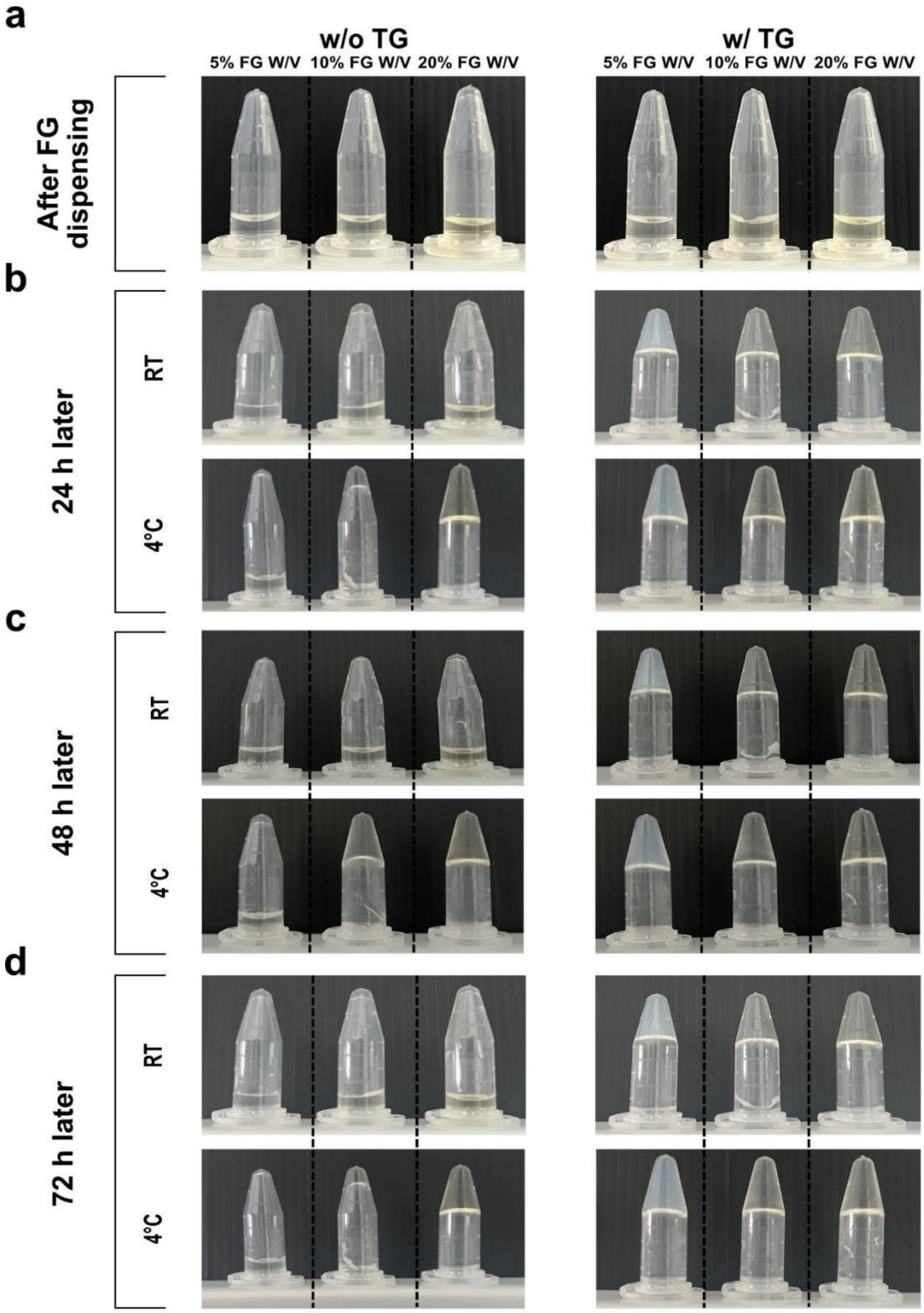

Supplementary Fig. S1. Detailed tube inversion analysis. Photographic images (cross-sectional view) of FG mixtures in Eppendorf tubes with and without TG at room temperature (RT) and 4°C. Snapshots were taken (**a**) right after mixing, and every 24 h for 72 h **(b-c-d)**.

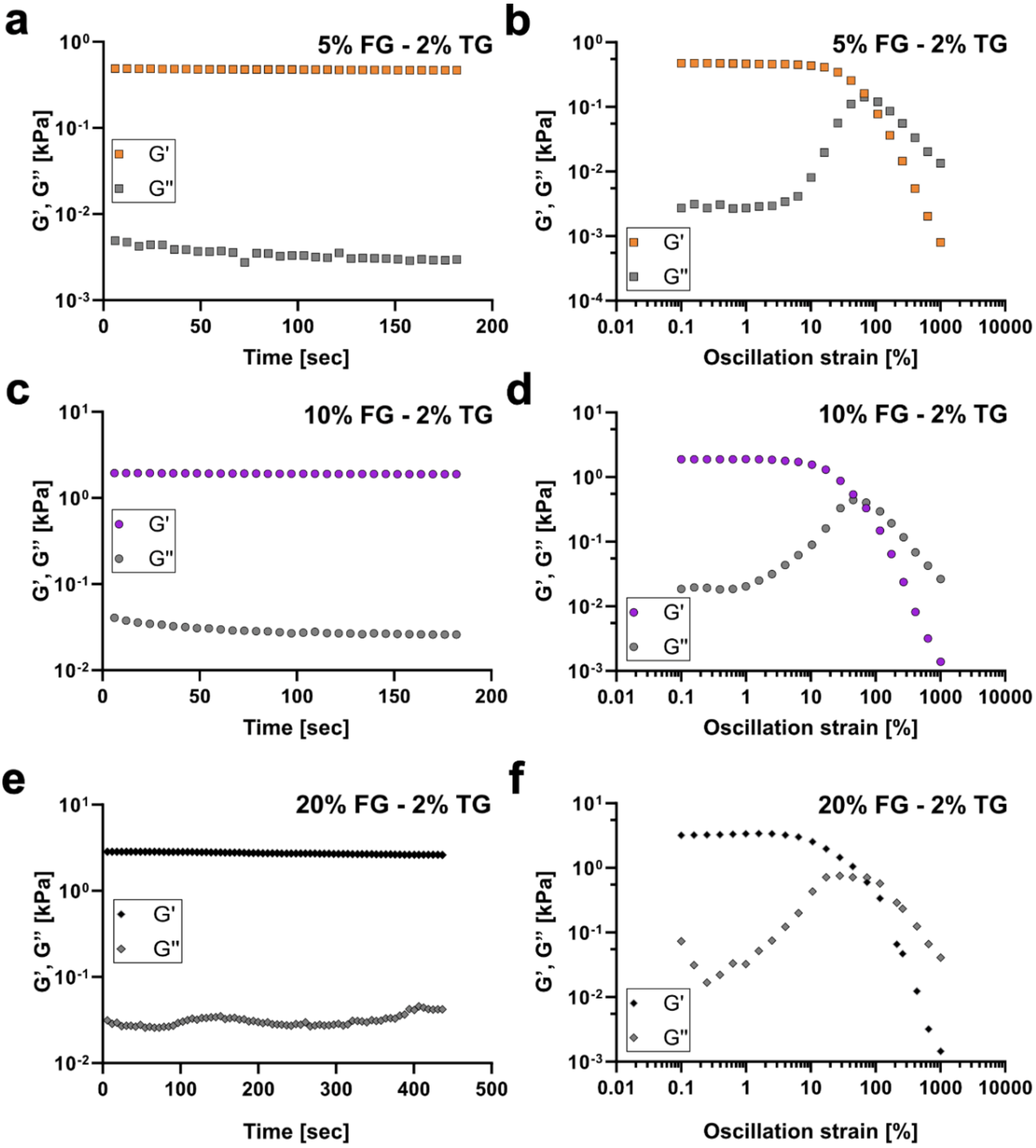

Supplementary Fig. S2. Fish gelatin hydrogels rheology. Three FG concentrations (**a-b** 5%, **c-d** 10%, **e-f** 20% w/v) were considered while the TG concentration was fixed at 2% w/v. Storage (G’) and Loss Modulus (G’’) vs. time (sec) and oscillation strain (%). Measurements were performed on n=3 independently prepared formulations and kept at 37 °C. Samples were prepared one day before the measurements, after overnight crosslinking at 37 °C.

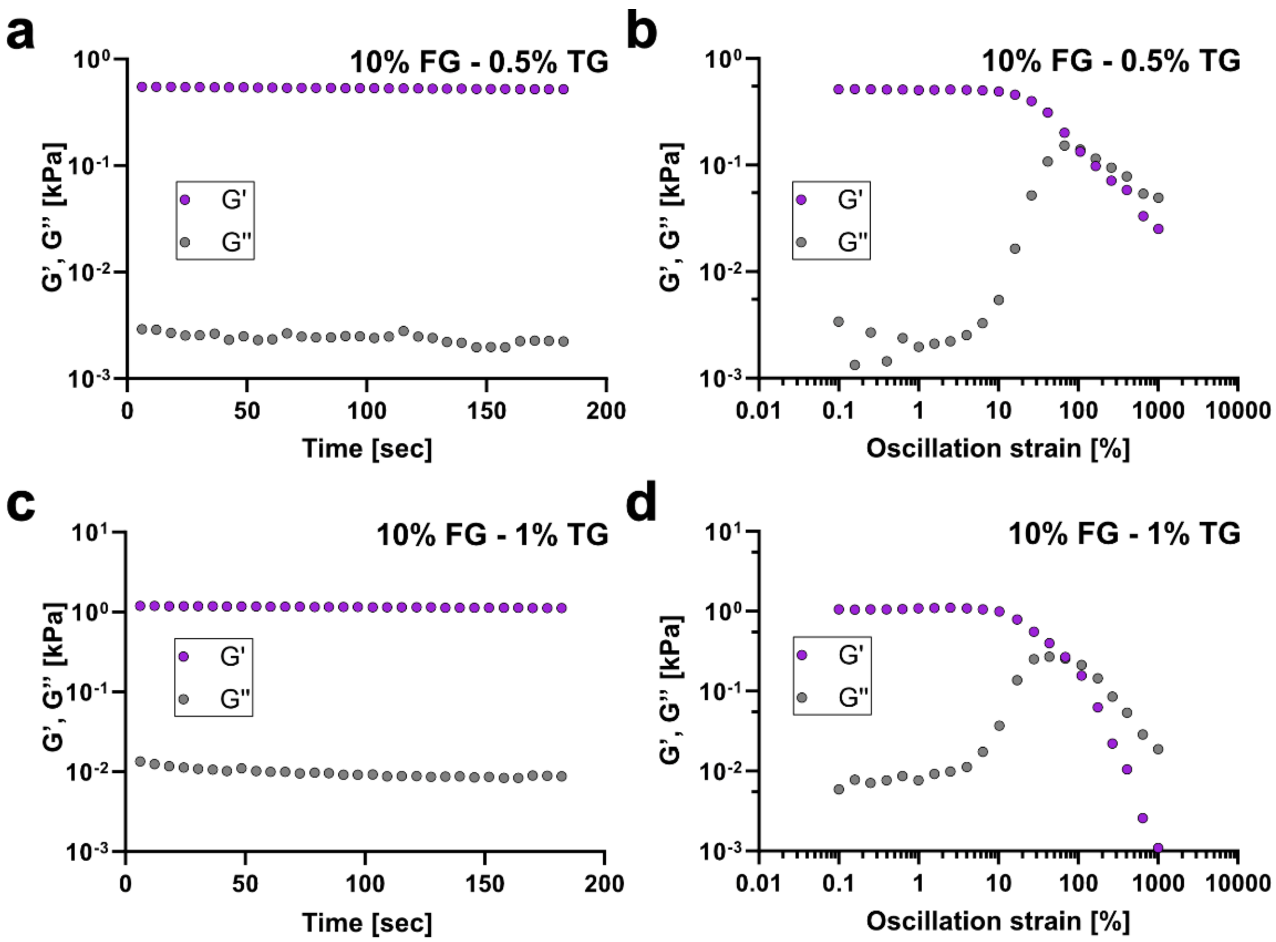

Supplementary Fig. S3. Fish gelatin hydrogels rheology. Two TG concentrations (**a-b** 0.5%, **c-d** 1%) were considered while the FG concentration was fixed at 10% w/v. Storage (G') and Loss Modulus (G'') vs. time (sec) and oscillation strain (%). Measurements were performed on n=3 independently prepared formulations and kept at 37 °C. Samples were prepared one day before the measurements, after overnight crosslinking at 37 °C.

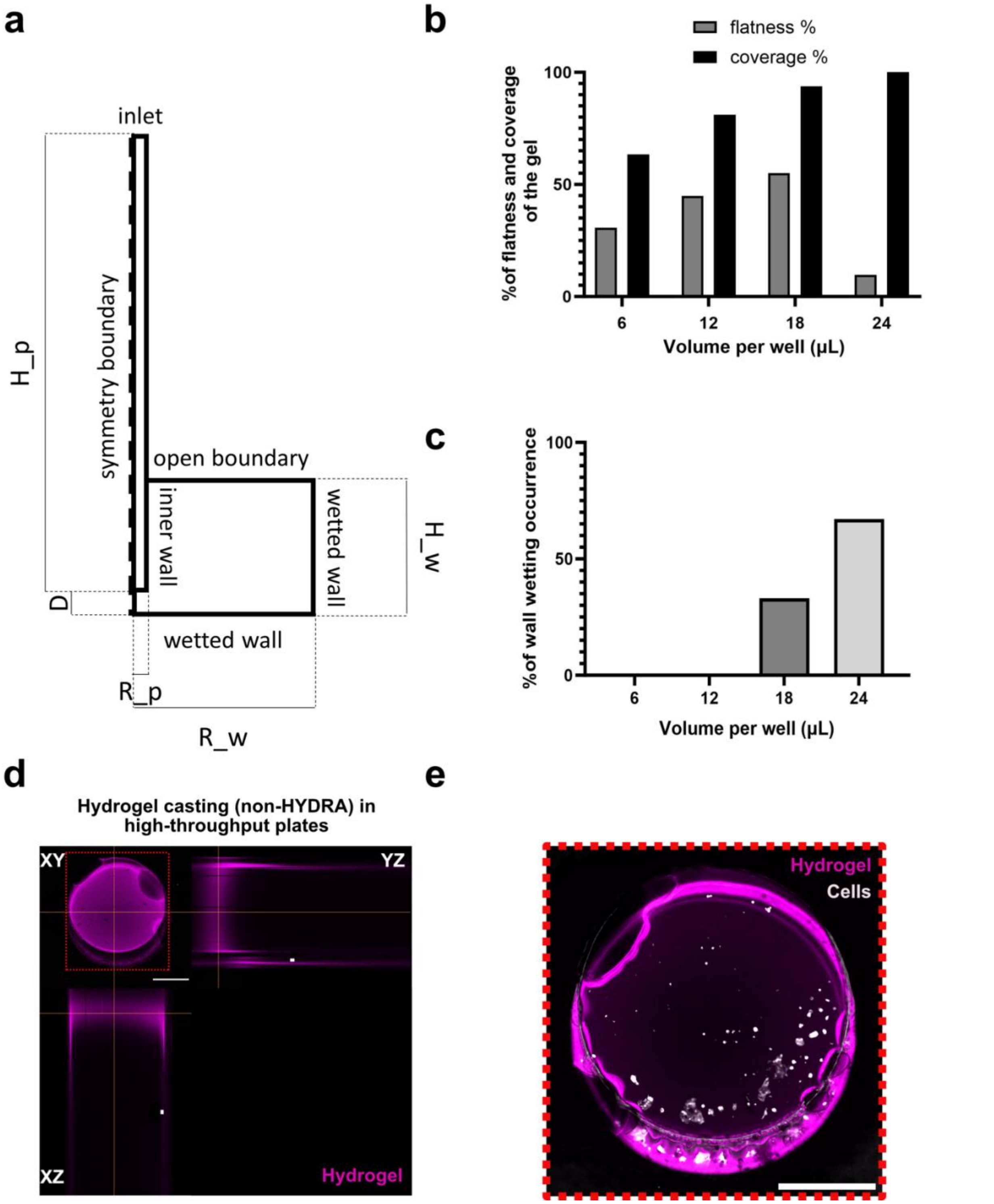

Supplementary Fig. S4. Modelling of the HYDRA dispensing method. (**a**) Schematic representation of the geometry and boundary conditions of the COMSOL model. A 2D axisymmetric model was chosen to save computational power and time. The well is modelled as a rectangle of dimensions R_w and H_w, and the pipette is modelled as a rectangle of dimensions R_p and H_p. An inlet boundary condition is applied at the upper edge of the pipette, and an open boundary condition is applied at the upper edge of the well since it is open. Two wet wall boundary conditions are applied at the two walls of the well. (**b**) Flatness and coverage percentages of the gel concerning the radius of the well, evaluated for different dispensed volumes (6 µl, 12 µl, 18 µl, and 24 µl), using computer modelling. (**c**) The occurrence of wall wetting for different volumes using COMSOL simulations. For each nominal volume, nine combinations of values were considered, namely accounting for small variations of volume (±20%) and well radius (-100 µm, and -200 µm, which are respectively the single contributions of plate tolerance dimension and robot calibration, and the sum of them). The value of wall-wetting occurrence is calculated as the ratio of combinations of values in the sweep leading to wall-wetting. (d-e) Hydrogels obtained by conventional liquid casting, displaying high curvature at well edges in contrast with meniscus-free hydrogels produced using HYDRA. (d) Representative z-stack reconstruction of a hydrogel with a full meniscus inside a high-throughput well, displayed in orthogonal views (xy, xz, yz). The hydrogel containing fluorescent beads is shown in magenta, while cells (actin) appear in gray. (e) Zoom-in of the xy plane from panel d, highlighting the curved meniscus across the entire well area.

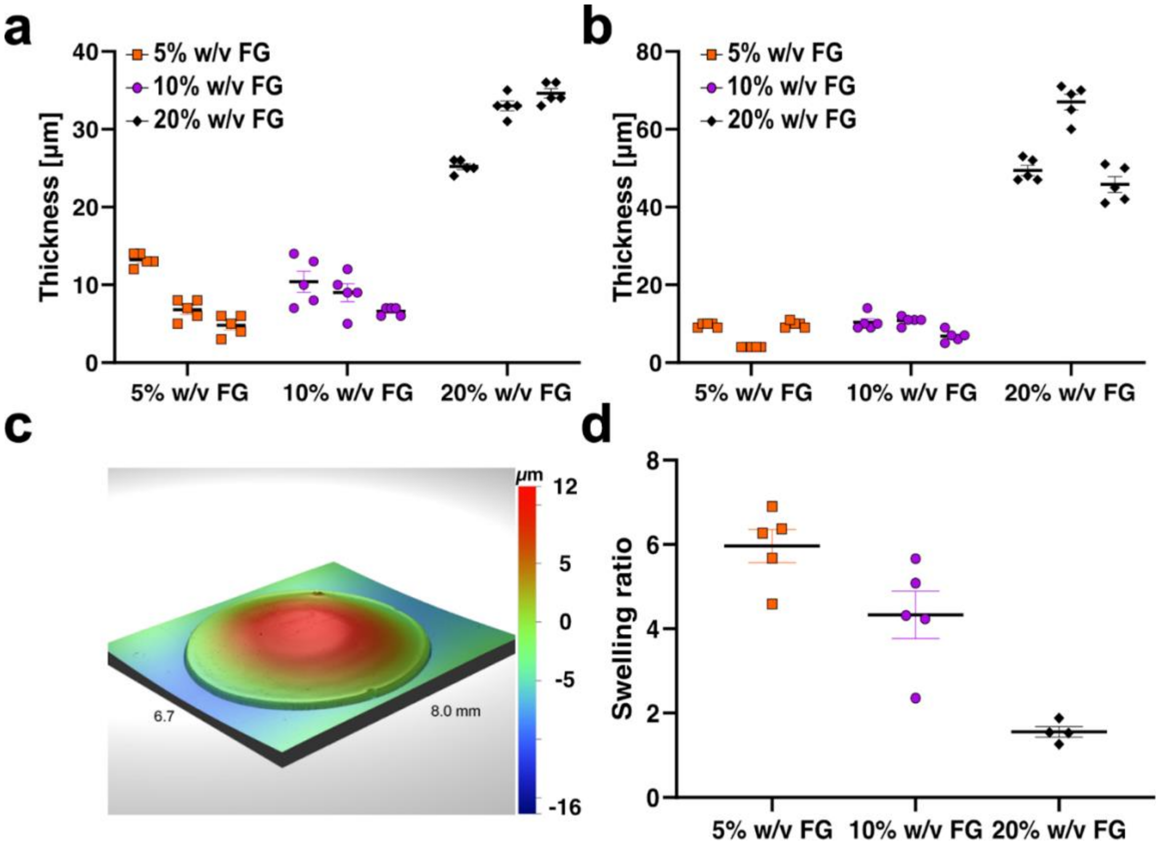

Supplementary Fig. S5. Assessment of thickness, flatness, and swelling of fish gelatin (FG) hydrogels. The graphs show two independent experiments (a–b) on hydrogel heights in the hydrated state (PBS 1×). Gel heights were calculated as the difference between the gel top (referenced using embedded fluorescent beads) and the gel bottom (referenced using HaCaT cell actin on plastic). Images were obtained using confocal imaging. Three different concentrations were tested (5% – orange squares, 10% – purple dots, and 20% w/v – black diamonds). The data correspond to single experiments (3 concentrations, n = 5 samples per concentration). For each sample, 5 FOVs were taken to measure the z-position and calculate thickness. Data are presented as mean ± s.e.m. (c) Representative 3D optical profilometry scan of a dry FG hydrogel showing surface topography and flatness. (d) Swelling ratios (h_wet/h_dry) calculated from hydrated and dry thickness measurements. Higher FG concentrations reduce swelling capacity, with ratios decreasing from ~6.7 (5% FG) to ~4.1 (10% FG) and ~1.1 (20% FG). Data are presented as mean ± s.e.m.

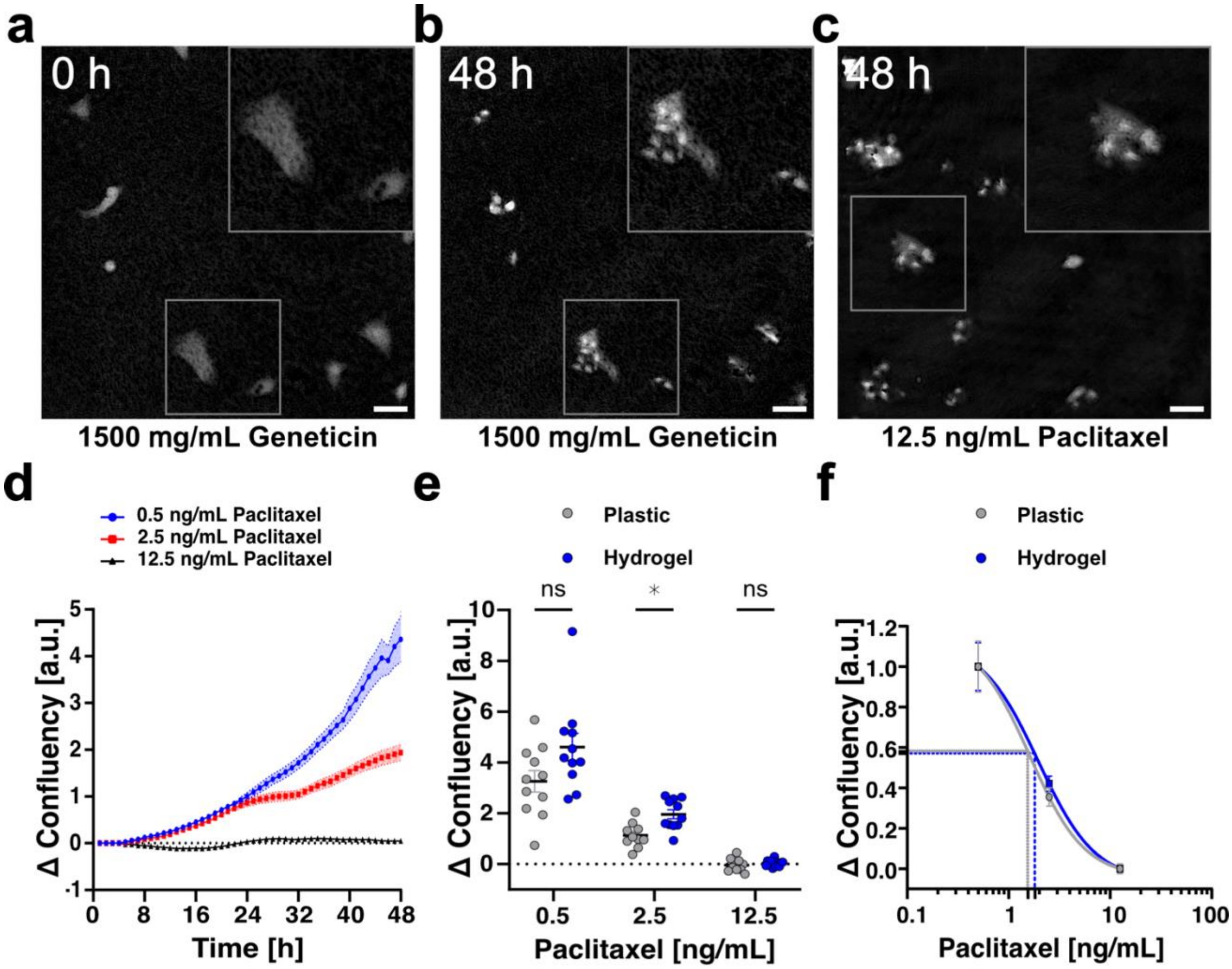

Supplementary Fig. S6. Drug test analysis using phase holographic imaging on hydrogel substrates. (a) Holographic image of HaCaT after seeding (1500 µg mL^{-1} - positive control) and (**b**) 48 h after seeding. (**c**) Holographic image of HaCaT treated with 12.5 ng mL^{-1} of paclitaxel drug 48 h after seeding. Scale bars: 50 µm. (**d**) HaCaT cell confluency vs. time. Cells were cultured in paclitaxel-based cell culture media (0.5 ng mL^{-1} – blue dots, 2.5 ng mL^{-1} – red squares, 12.5 ng mL^{-1} black triangles) for 48 h on hydrogel substrates. Data were normalized concerning the initial confluency value. Solid lines represent mean values, and shaded areas represent s.e.m. (n=12). (**e**) Final cell confluency vs. paclitaxel concentration (0.5 ng mL^{-1}, 2.5 ng mL^{-1}, 12.5 ng mL^{-1}) on plastic and hydrogel substrates. Data were normalized concerning the initial confluency value. (*) stands for significative difference. (**f**) Paclitaxel dose-response curves on plastic (IC50, 1.6 ng mL^{-1}) and hydrogel (IC50, 1.8 ng mL^{-1}) substrates (n=12). To help visualize the half-maximal inhibitory concentration (IC50), horizontal and vertical dotted lines mark the intercepts on the Y- and X-axes, respectively, corresponding to the IC50 values for each condition. Data were normalized concerning the initial confluency value (n=12). Image brightness and contrast were adjusted for printed visibility. Data are presented as mean ± s.e.m.

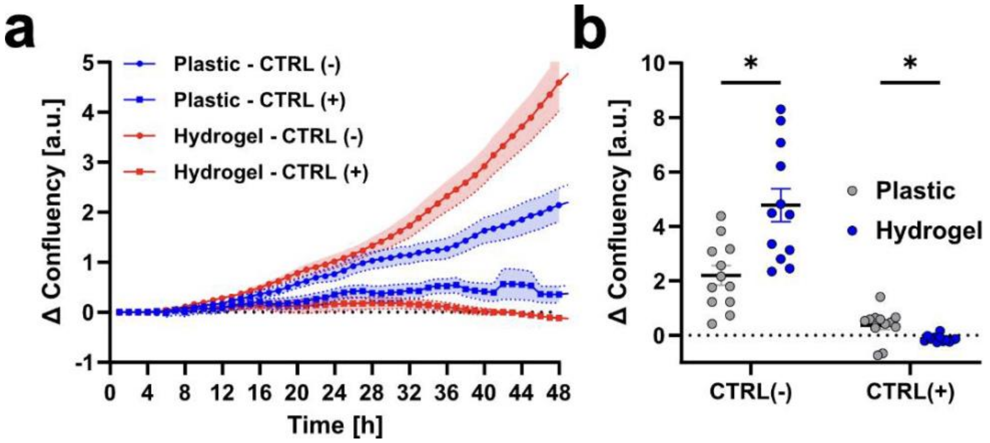

Supplementary Fig. S7. Control (CTRL) data from holographic imaging of nocodazole- and paclitaxel-treated cells on hydrogel and plastic. (**a-b**) HaCaT cell confluency vs. time. Cells were cultured in 0.1% DMSO (CTRL (-)) or 1500 µg mL^{-1} Geneticin (CTRL (+)) on both hydrogel and plastic substrates for 48 h. Data were normalized concerning the initial confluency value. Solid lines represent mean values, and shaded areas represent s.e.m. (n=12). **B)** Final cell confluency on plastic and hydrogel substrates. Data were normalized concerning the initial confluency value (n=12). (*) stands for significative difference.

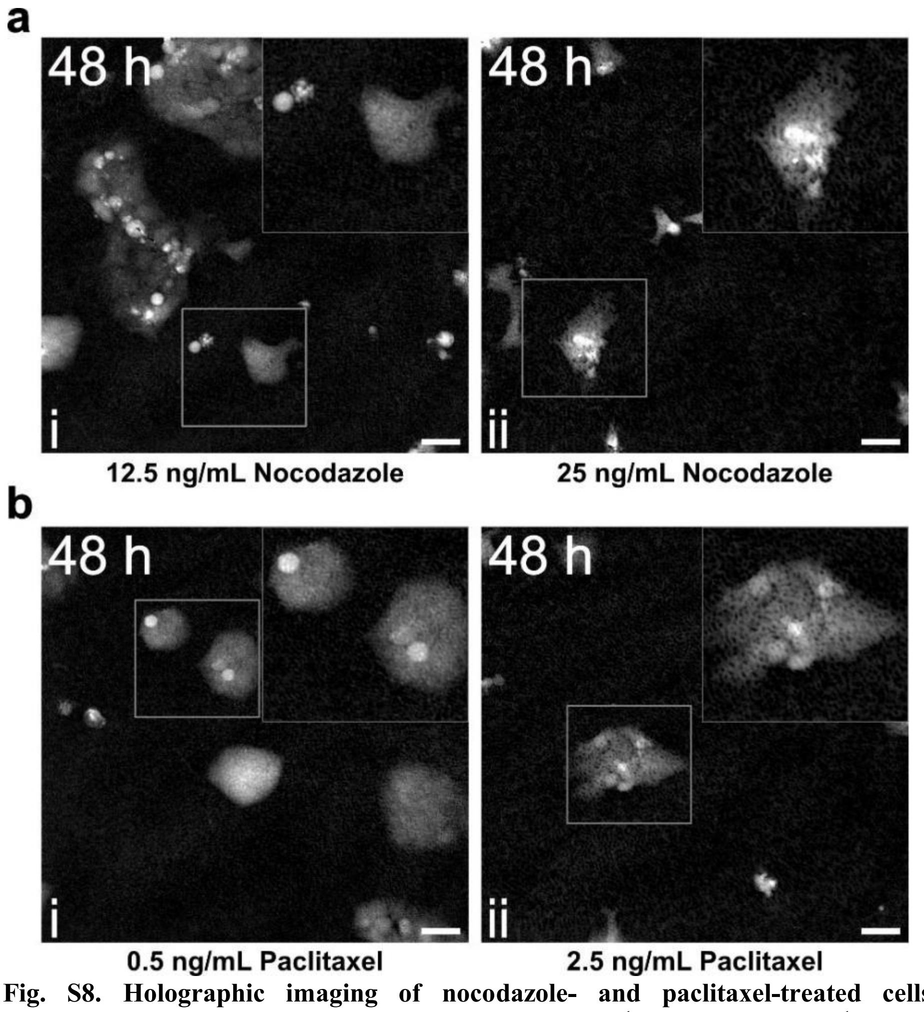

Supplementary Fig. S8. Holographic imaging of nocodazole- and paclitaxel-treated cells on hydrogel substrates. (a) Holographic image of HaCaT treated with **(i)** 12.5 ng mL^{-1} and **(ii)** 25 ng mL^{-1} of nocodazole drug 48 h after seeding. (**b**) Holographic image of HaCaT treated with **(i)** 0.5 ng mL^{-1} and **(ii)** 2.5 ng mL^{-1} of paclitaxel drug 48 h after seeding. Image brightness and contrast were adjusted for printed visibility.

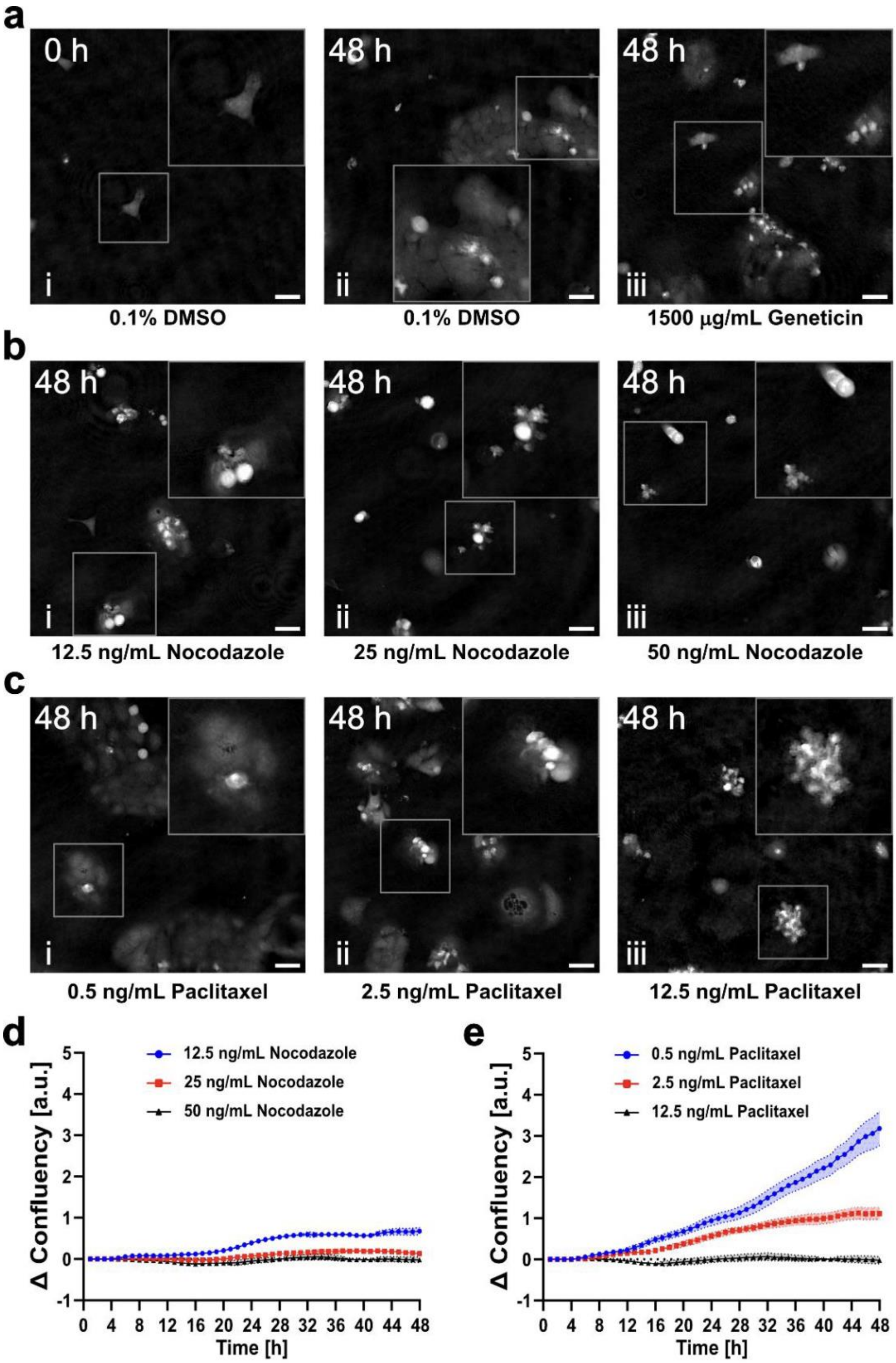

Supplementary Fig. S9. Holographic imaging analysis of nocodazole- and paclitaxel-treated cells on plastic substrates. (a) Holographic image of HaCaT **(i)** after seeding (0.1% DMSO – vehicle negative control) and **(ii)** 48 h after seeding. **(iii)** Holographic image of HaCaT (1500 μg mL^{-1} - positive control) 48 h after seeding. (**b**) Holographic image of HaCaT treated with **(i)** 12.5 ng mL^{-1}, **(ii)** 25 ng mL^{-1}, **(iii)** and 50 ng mL^{-1} of nocodazole drug, 48 h after seeding. (**c**) Holographic image of HaCaT treated with **(i)** 0.5 ng mL^{-1}, **(ii)** 2.5 ng mL^{-1}, **(iii)** and 12.5 ng mL^{-1} of paclitaxel drug 48 h after seeding. (**d**) HaCaT cell confluency vs. time. Cells were cultured in nocodazole-based (12.5 ng mL^{-1} – blue dots, 25 ng mL^{-1} – red squares, 50 ng mL^{-1} black triangles) or (**e**) paclitaxel-based cell culture media (0.5 ng mL^{-1} – blue dots, 2.5 ng mL^{-1} – red squares, 12.5 ng mL^{-1} black triangles) for 48 h on plastic substrates. Data were normalized concerning the initial confluency value. Solid lines represent mean values, and shaded areas represent s.e.m. (n=12). Image brightness and contrast were adjusted for printed visibility.

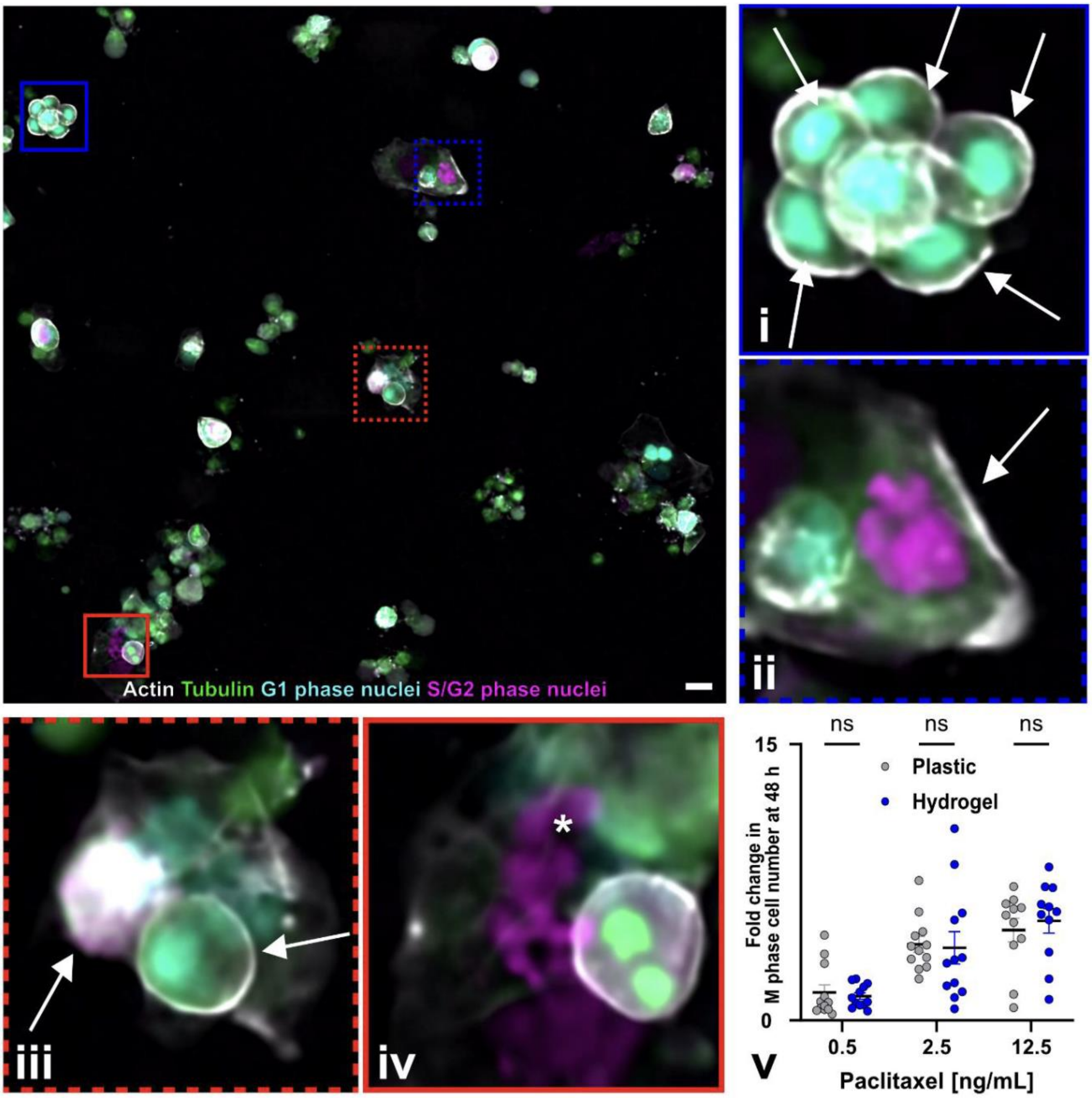

Supplementary Fig. S10. Drug test using fluorescence imaging on HYDRA hydrogels cast on inexpensive traditional tissue culture plastic. Static widefield images of HaCaT (RFP – Life Act, in gray, GFP – tagged tubulin, in green) 48 h after seeding. Cells were treated with 12.5 ng mL^{-1} paclitaxel. Arrows stand for **(i)** Cells in the G1 phase (in cyan), **(ii)** cells in the S/G2 phase (in magenta), and **(iii)** cells in the mitotic (M) phase. The asterisk stands for **(iv)** nuclear fragmentation. **(v)** Fold change in M phase cell number after 48 h vs. paclitaxel concentration on plastic and hydrogel substrates. Cell number in the M phase was counted as a fraction of the total cell number in a FOV and then normalized to the vehicle negative control (n=12). Data are displayed as mean ± s.e.m. (ns) stands for no statistical difference. Images were taken on hydrogel thin layers cast on plastic plates. Scale bar: 25 μm.

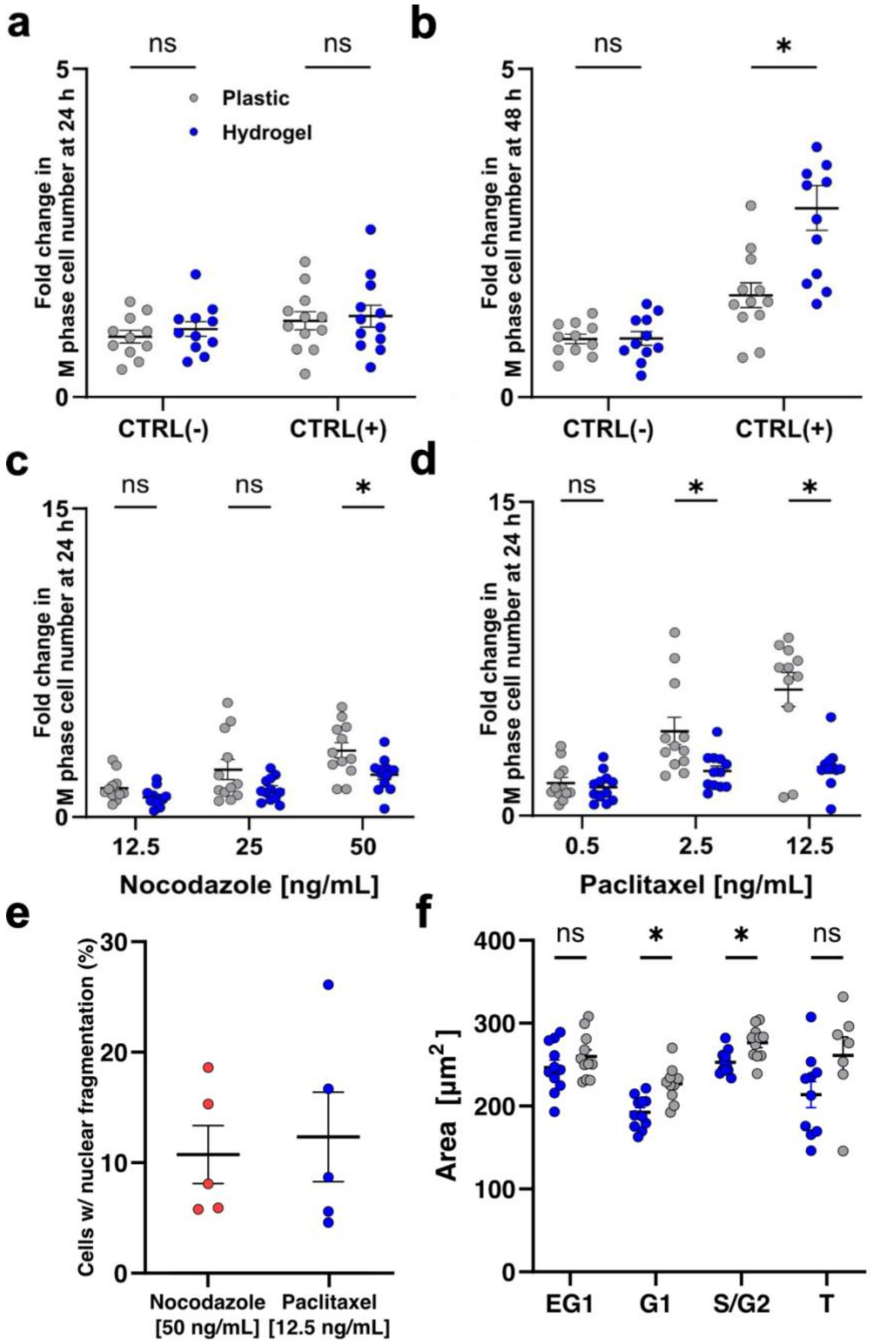

Supplementary Fig. S11. Static fluorescence imaging analysis of HYDRA hydrogels cast on inexpensive traditional tissue culture plastic. Fold change in M phase cell number after 48 h vs. (**a-b**) negative and positive controls, (**c**) nocodazole, (**d**) paclitaxel concentration on plastic and hydrogel substrates. Cell number in the M phase was counted as a fraction of the total cell number in a FOV and then normalized to the vehicle negative control (n=12). Data are displayed as mean ± s.e.m. (ns) stands for no statistical difference. (**e**) Manual counts of cells exhibiting nuclear fragmentation were performed across five independent fields of view (FOVs) for each condition. Bars represent the mean percentage of cells with nuclear fragmentation (± s.e.m.) relative to the total cell population, while individual dots indicate values from each FOV. On average, ~11% of cells treated with nocodazole (50 ng/mL) and ~12% of cells treated with paclitaxel (12.5 ng/mL) displayed nuclear fragmentation and were excluded from subsequent analyses. (**f**) Area of the nuclei was quantified under control conditions (0.1% DMSO, i.e. negative control) in cells cultured on plastic (grey) and hydrogel (blue) substrates and stratified by cell-cycle phase (EG1, G1, S/G2, T). Measurements were taken 48 h after seeding. Cells grown on plastic displayed significantly larger projected nuclear areas in G1 and S/G2 compared to hydrogel ($p < 0.05$), while no significant differences were observed in EG1 and T. Each dot represents the mean area value in each well; horizontal bars indicate mean ± SEM.

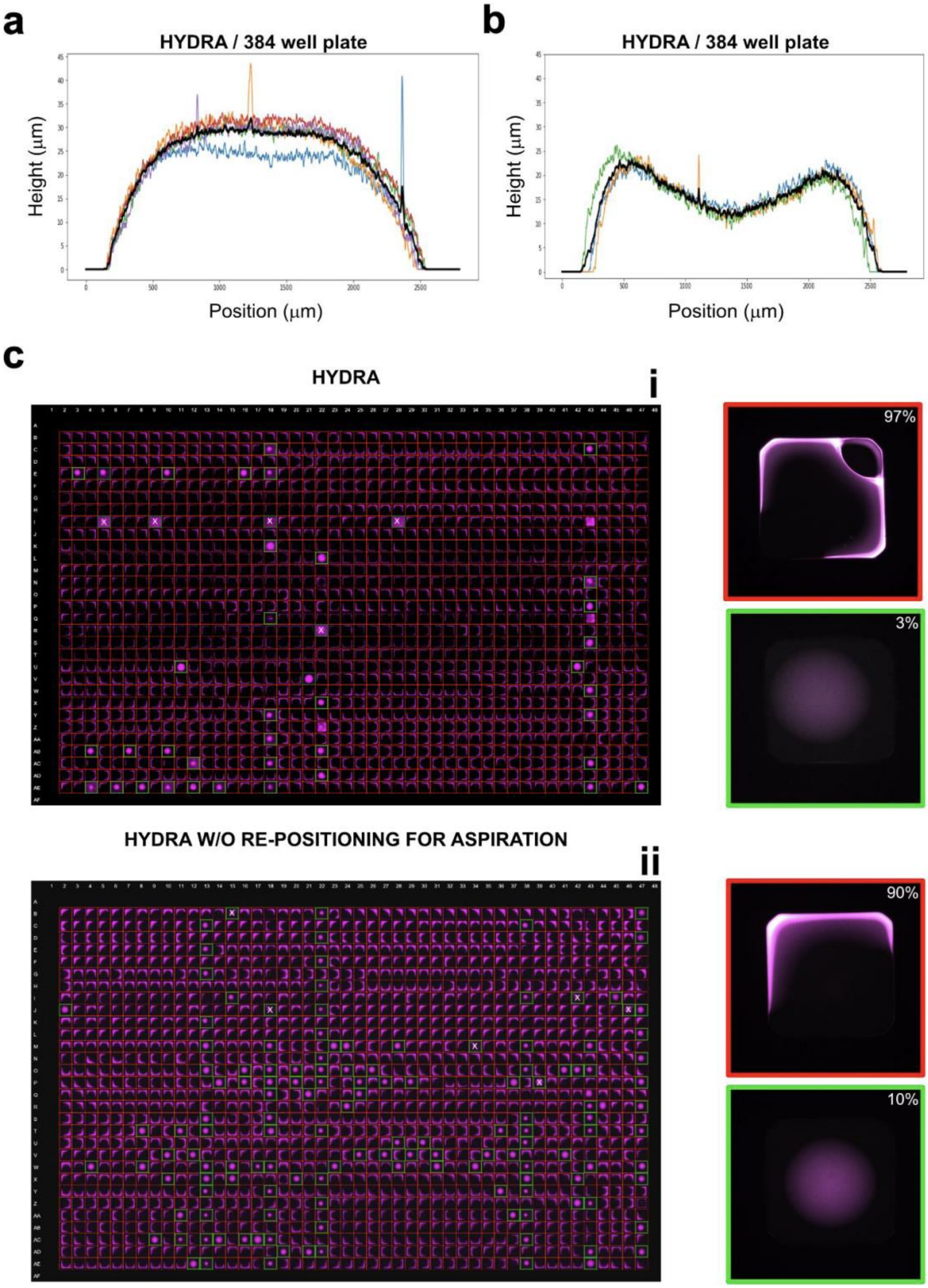

Supplementary Fig. S12. HYDRA-like hydrogels on 384- and 1536-well plates. (**a**-**b**) Profile reconstructions of hydrogel samples in a 384-well plate showing two main populations: (**a**) one with a planar shape and (**b**) another with a concave shape. (**c**) HYDRA performed on a 1536-well plate. **i)** Using the HYDRA method, the hydrogels contact the well walls in almost the entire plate (97%) because the minimum liquid handling volume is too high for the well diameter. **ii)** Since robot movement between dispensing and aspiration could be a factor, if HYDRA is performed without repositioning for aspiration, the success rate increases slightly (from 3% to 10%). “x” symbols on the well show wrong classification done by the automatic analysis.

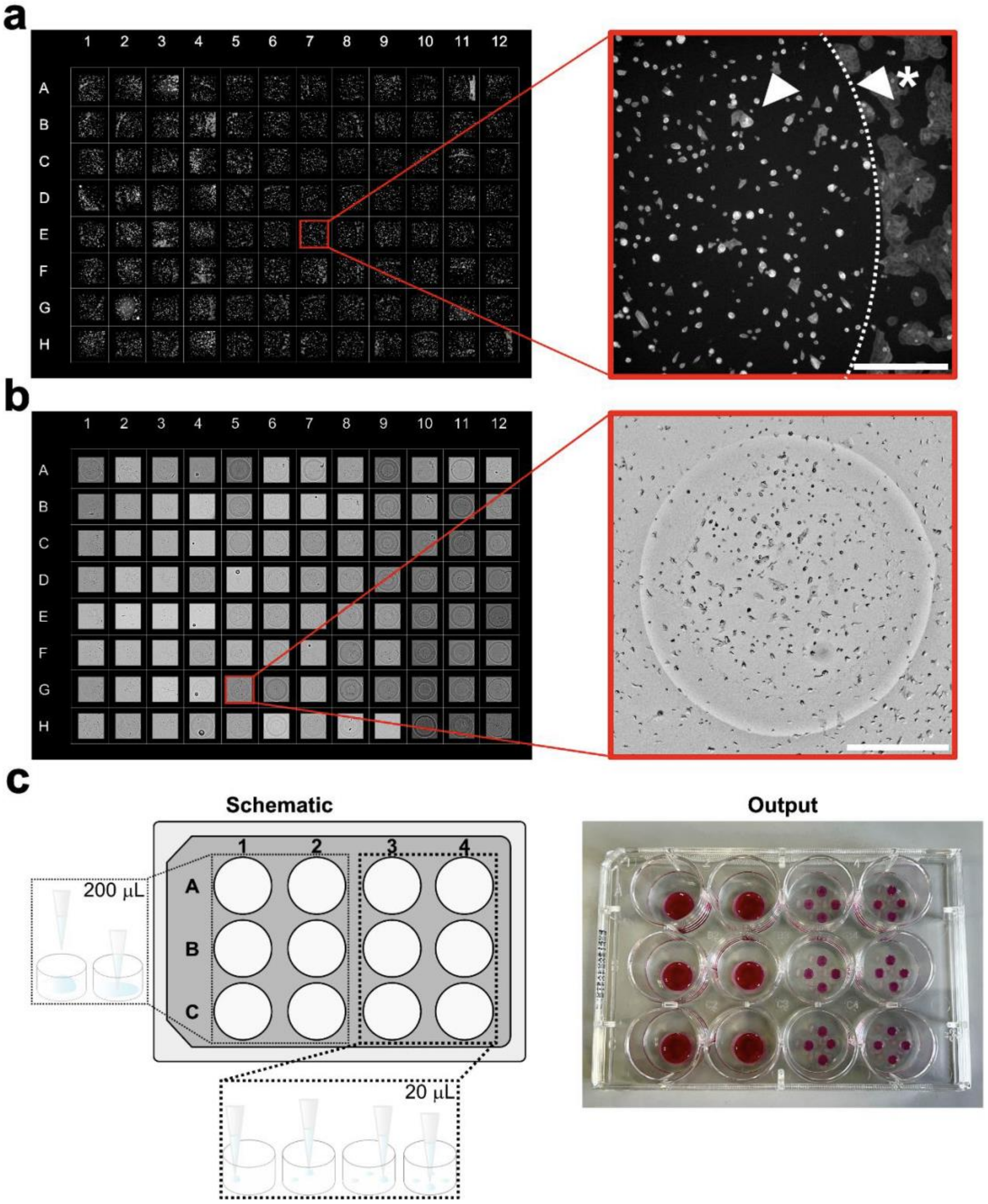

Supplementary Fig. S13. Further applications of HYDRA-like gels. (**a**) 96-well plate tiling of HaCaT cells (actin in gray) seeded on hydrogels that were stored for two months in PBS at 4°C. Images were acquired with a 10x air objective (NA 0.3). Less than half of the hydrogel was captured (white arrow). A dashed line separates the hydrogel from the plastic substrate (white arrow with an asterisk). Scale bar: 500 µm. (**b**) 96-well plate tiling of HaCaT cells on hydrogels attached to glass. The plate with a glass bottom was first automatically functionalized using the OT-2 robot from Opentrons under the chemical hood, and then HYDRA was applied to cast the gels on the functionalized surface. To acquire the entire hydrogel with a 4x air objective (NA 0.13), 2 µL was used to cast the gels. Scale bar: 1 mm. (**c**) The HYDRA method can also be used in low-throughput plates. A schematic shows the custom protocol made using the OT-2 robot from Opentrons and a 12-well plate. The robot cast HYDRA-like hydrogels with a large volume (200 µL) in the first half of the plate, while increasing throughput in the second half by fabricating 4 HYDRA-like gels (20 µL each), which are 10 times smaller than the others. The output image shows the entire plate with hydrogel precursor solution pre-mixed with red food coloring (2 µL left to visualize the gels in this experiment).

Supplementary Table S1. Hydrogel-based plate formats: throughput, imaging compatibility, strengths, and limitations.

Platform	Typical Throughput	Hydrogel Thickness / Geometry	Imaging Compatibility	Primary Use / Strength	Key Limitations
HYDRA (this work)	96–384 wells	10–50 µm flat film (meniscus-free)	High-content imaging compatible	High-throughput 2D soft-substrate assays; imaging-optimized	No encapsulation (2D only)
RASTRUM (Inventia)[1]	96–384 wells	200–1000 µm printed 3D microtissues	Confocal/3D imaging	3D cell-laden bioprinting for drug response	Thick constructs; low optical clarity for 2D imaging
TrueGel3D HTS (Merck)[2]	96 wells	500–1000 µm bulk PEG gels	Confocal/3D imaging	3D invasion/viability assays	Meniscus curvature; poor single-plane imaging
PEG-based biomaterials (Brooks 2018)[3]	96 wells	500-1000 µm	Confocal/3D imaging	Live/Dead Cell viability assays	Surface imaging limited by thickness
LbL polyelectrolyte films (Machillot 2018)[4]	96 wells	10 nm–1 µm	Excellent optical flatness	Surface chemistry tuning; mechanobiology	Too thin to mask stiff substrate
LM-Well insert (Milton 2025)[5]	96 wells (multi-niche)	Multiple small flat gels	Imaging-friendly regions	Co-culture or organoid interface studies	Manual insert handling; moderate throughput
2D/3D Hydrogel platforms (Skelton 2024)[6]	96 wells	100–300 µm molded gels	Compatible with plate readers & high-content imaging	Multiplexed viscoelastic matrices	Assembled plate; moderate automation
LigHTS (Enrico 2025)[7]	384–1536 wells	10–60 µm photopatterned GelMA	High-content imaging compatible	Ultra-HTS patterned hydrogel coatings	Specialized photochemistry equipment

Supplementary Table S2. Parameters used in the COMSOL model.

Parameter	Value	Unit
R_w	3.45	[mm]
H_w	3	[mm]
R_p	0.16	[mm]
H_p	230	[mm]
D	0.3	[mm]
Theta_adv	40	[°]
Theta_rec	10	[°]

Supplementary References

1. Utama, R. H. *et al.* A 3D Bioprinter Specifically Designed for the High-Throughput Production of Matrix-Embedded Multicellular Spheroids. *iScience* **23**, (2020).
2. Zhang, N. *et al.* Soft Hydrogels Featuring In-Depth Surface Density Gradients for the Simple Establishment of 3D Tissue Models for Screening Applications. *SLAS Discovery* **22**, 635–644 (2017).
3. Brooks, E. A., Jansen, L. E., Gencoglu, M. F., Yurkevicz, A. M. & Peyton, S. R. Complementary, Semiautomated Methods for Creating Multidimensional PEG-Based Biomaterials. *ACS Biomater. Sci. Eng.* **4**, 707–718 (2018).
4. Machillot, P. *et al.* Automated Buildup of Biomimetic Films in Cell Culture Microplates for High-Throughput Screening of Cellular Behaviors. *Advanced Materials* **30**, 1801097 (2018).
5. Milton, L. A. *et al.* Building multiple microenvironmental niches using a customizable 3D printed well insert. *Lab Chip* (2025) doi:10.1039/D5LC00753D.
6. Skelton, M. L. *et al.* Modular Multiwell Viscoelastic Hydrogel Platform for Two- and Three-Dimensional Cell Culture Applications. *ACS Biomater. Sci. Eng.* **10**, 3280–3292 (2024).
7. Enrico, A. *et al.* LigHTS: Massively Parallel Biomimetic Photo-Functionalization for Imaging-Based Ultra-High-Throughput Screening. *bioRxiv* https://doi.org/10.1101/2025.10.23.683892 (2025) doi:10.1101/2025.10.23.683892.
