# Supplementary material for: Fabrication of cell culture hydrogels by robotic liquid handling automation for high-throughput drug testing
Source: Commun Eng. 2025 Dec 22;4:222. doi: 10.1038/s44172-025-00575-3 (PMC12749810; doi:10.1038/s44172-025-00575-3)
Supplement: Supplementary file 3 — Description of Additional Supplementary Files [file 44172_2025_575_MOESM3_ESM.pdf]

Description of Additional Supplementary Files:

File: Supplementary Movie S1

Description: Phase field simulation of large volume dispensing with meniscus formation. Simulation of dispensing of a large volume of hydrogel precursor solution that touches the walls and forms the classic meniscus, using the 'Laminar Two-phase Flow, Phase Field" interface in COMSOL. In this Supplementary Movie, there is both the volume fraction of fluid (on the left) and the profile angle (on the right) visualization. In the volume fraction of fluid visualization, the two colors (blue and red) are used to represent the two different fluids, respectively hydrogel precursor solution and air, and the transition between the two colors represents the interface between the fluids. In the profile angle visualization, the colors represent the angle of the dispensed liquid at each point of the interface between the hydrogel precursor solution and air

File: : Supplementary Movie S2

Description: **Phase field simulation of HYDRA dispensing method**. Simulation of dispensing and reaspiration of 12 µl of hydrogel precursor solution, using the 'Laminar Two-phase Flow, Phase Field" interface in COMSOL. In this Supplementary Movie, there is both the volume fraction of fluid (on the left) and the profile angle (on the right) visualization. In the volume fraction of fluid visualization, the two colors (blue and red) are used to represent the two different fluids, respectively hydrogel precursor solution and air, and the transition between the two colors represents the interface between the fluids. In the profile angle visualization, the colors represent the angle of the dispensed liquid at each point of the interface between the hydrogel precursor solution and air.

File: : Supplementary Movie S3

Description: **Automated fabrication of hydrogel thin films in high throughput using a liquid handling system**. The Supplementary Movie shows the HYDRA method we use to cast hydrogel thin films in a 96- well plate by using a robot from Integra. The first protocol starts by mixing two solutions placed in the tube rack (on the right side of the field of view). Then, a second protocol uses the pre-mixed solution (in red) prepared before to dispense and re-aspirate the gels along columns, by placing the robot tip near the bottom of the 96-well plate (<=100 µm). 20% w/v fish gelatin solution (with a red colorant) and 4% w/v

transglutaminase were used in this Supplementary Movie. A volume of 1 μL of solution was intentionally retained to enhance the visualization of the hydrogel layers.

File: Supplementary Movie S4

Description: Automated fabrication of hydrogel thin films in high throughput using a liquid handling system: zoom on a single well of a 96-well plate. The Supplementary Movie shows a close-up of a well in the 96-well plate using the HYDRA method. The pre-mixed hydrogel solution (in red) is dispensed and re-aspirated in the well, by placing the robot tip near the bottom of the 96- well plate (<=100 μm). 20% w/v fish gelatin solution (with a red colorant) and 4% w/v transglutaminase pre-mixed solution were used in this Supplementary Movie. A volume of 1 μL of solution was intentionally retained to enhance the visualization of the hydrogel layer.

File: Supplementary Movie S5

Description: Automated holographic imaging of cells on hydrogel substrate. The Supplementary Movie shows a series of 8 time-lapse sequences of the 96-well plate utilized for conducting a drug test. Each of the 8 fields of view (FOVs) corresponds to a specific condition of the drug test conducted on hydrogel substrates. These conditions are, as follows: the vehicle negative control (0.1% DMSO), positive control (1500 μg mL-1 geneticin), nocodazole concentrations of 12.5 ng mL-1, 25 ng mL-1, and 50 ng mL-1, as well as paclitaxel concentrations of 0.5 ng mL-1, 2.5 ng mL-1, and 12.5 ng mL-1. Scale bar: 50 μm.

File: Supplementary Movie S6

Description: Automated holographic imaging of cells on a plastic substrate. The Supplementary Movie shows a series of 8 time-lapse sequences of the 96-well plate utilized for conducting a drug test. Each of the 8 fields of view (FOVs) corresponds to a specific condition of the drug test conducted on plastic substrates. These conditions are, as follows: the vehicle negative control (0.1% DMSO), positive control (1500 μg mL-1 geneticin), nocodazole concentrations of 12.5 ng mL-1, 25 ng mL-1, and 50 ng mL-1, as well as paclitaxel concentrations of 0.5 ng mL-1, 2.5 ng mL-1, and 12.5 ng mL-1. Scale bar: 50 μm.

File: Supplementary Movie S7

Description: HTS plate automatic acquisition. The Supplementary Movie demonstrates the automated acquisition of HYDRA-like gels in a 384-well plate, with each field of view (FOV) representing a single well. This automated process is created using a Nikon JOB script and a 4X air objective (NA 0.13). For each well, the microscope moves to the center and captures a multi-channel image: one channel visualizes fluorescent beads embedded in the gel, and the other visualizes the well walls. This acquisition pipeline can be applied to every HTS plate by changing the corresponding plate in the script. The Supplementary Movie is accelerated by a factor of 10.

File: Supplementary Movie S8

Description: HTS plate analysis pipeline. The Supplementary Movie shows the automatic quality control analysis of hydrogels in an HTS plate. The ImageJ macro script uses two channels acquired during the automatic acquisition: one visualizing beads embedded in the gel and the other visualizing the well walls. The script separates the two channels, applies thresholding, and generates a final composite image using a logical AND operation of the thresholded images. This composite image determines if the gels have contacted the walls. Additionally, the mean intensity of the mask obtained from the gel indicates whether the gel is planar or concave.

File: Supplementary Movie S9

Description: Automated long-term live fluorescence imaging on hydrogels. The Supplementary Movie displays a long-term live fluorescence imaging of a cluster of HaCaT cells, genetically engineered to express fluorescent markers for Actin and our novel cell cycle sensor, FUCCIplex. In the Supplementary Movie, actin is visualized in gray, and nuclei, depending on their cell cycle phase, vary between cyan (G1 phase) and magenta (S/G2/M phase). The experiment was conducted using a 40X silicon oil objective (NA 1.25) with perfect focus on overtime. Images were taken every 15 min for 18 h. Scale bar: 25 µm.
