# Supplementary material for: Fabrication of cell culture hydrogels by robotic liquid handling automation for high-throughput drug testing
Source: Commun Eng. 2025 Dec 22;4:222. doi: 10.1038/s44172-025-00575-3 (PMC12749810; doi:10.1038/s44172-025-00575-3)
Supplement: Supplementary file 13 — Reporting Summary [file 44172_2025_575_MOESM13_ESM.pdf]

nature portfolio

Corresponding author(s): Francesco Silvio Pasqualini, Alessandro Enrico

Last updated by author(s): Nov 11, 2025

Reporting Summary

Nature Portfolio wishes to improve the reproducibility of the work that we publish. This form provides structure for consistency and transparency in reporting. For further information on Nature Portfolio policies, see our Editorial Policies and the Editorial Policy Checklist.

Statistics

For all statistical analyses, confirm that the following items are present in the figure legend, table legend, main text, or Methods section.

n/a	Confirmed	
☐	☒	The exact sample size (*n*) for each experimental group/condition, given as a discrete number and unit of measurement
☐	☒	A statement on whether measurements were taken from distinct samples or whether the same sample was measured repeatedly
☐	☒	The statistical test(s) used AND whether they are one- or two-sided<br>*Only common tests should be described solely by name; describe more complex techniques in the Methods section.*
☒	☐	A description of all covariates tested
☐	☒	A description of any assumptions or corrections, such as tests of normality and adjustment for multiple comparisons
☐	☒	A full description of the statistical parameters including central tendency (e.g. means) or other basic estimates (e.g. regression coefficient) AND variation (e.g. standard deviation) or associated estimates of uncertainty (e.g. confidence intervals)
☐	☒	For null hypothesis testing, the test statistic (e.g. *F*, *t*, *r*) with confidence intervals, effect sizes, degrees of freedom and *P* value noted<br>*Give P values as exact values whenever suitable.*
☒	☐	For Bayesian analysis, information on the choice of priors and Markov chain Monte Carlo settings
☒	☐	For hierarchical and complex designs, identification of the appropriate level for tests and full reporting of outcomes
☒	☐	Estimates of effect sizes (e.g. Cohen's *d*, Pearson's *r*), indicating how they were calculated

Our web collection on statistics for biologists contains articles on many of the points above.

Software and code

Policy information about availability of computer code

Data collection	Nis package v. 6.10.02, HOLOMONITOR App Suite v. 4.0.0.536
Data analysis	Graphpad PRISM v. 10.6.1. Custom code is available at the following links: https://github.com/Synthetic-Physiology-Lab/fucciphase.git, https://github.com/Synthetic-Physiology-Lab/BioimageAnalysisCollection.git

For manuscripts utilizing custom algorithms or software that are central to the research but not yet described in published literature, software must be made available to editors and reviewers. We strongly encourage code deposition in a community repository (e.g. GitHub). See the Nature Portfolio guidelines for submitting code & software for further information.

Data

Policy information about availability of data

All manuscripts must include a data availability statement. This statement should provide the following information, where applicable:

- Accession codes, unique identifiers, or web links for publicly available datasets
- A description of any restrictions on data availability
- For clinical datasets or third party data, please ensure that the statement adheres to our policy

All data that support the findings of this study are included within the article (and any supplementary files).

Research involving human participants, their data, or biological material

Policy information about studies with human participants or human data. See also policy information about sex, gender (identity/presentation), and sexual orientation and race, ethnicity and racism.

Reporting on sex and gender	/
Reporting on race, ethnicity, or other socially relevant groupings	/
Population characteristics	/
Recruitment	/
Ethics oversight	/

Note that full information on the approval of the study protocol must also be provided in the manuscript.

Field-specific reporting

Please select the one below that is the best fit for your research. If you are not sure, read the appropriate sections before making your selection.

☒ Life sciences ☐ Behavioural & social sciences ☐ Ecological, evolutionary & environmental sciences

For a reference copy of the document with all sections, see nature.com/documents/nr-reporting-summary-flat.pdf

Life sciences study design

All studies must disclose on these points even when the disclosure is negative.

Sample size	Sample sizes varied between experiments depending on the assay type and imaging format. Quantitative analyses typically included three independent experimental groups with n = 3–5 samples per group, while high-throughput assays (e.g., condition or drug screens) included up to n = 12 replicates per condition. A minimum of three replicates was chosen for all quantitative analyses to enable statistical evaluation of variability and significance. No formal power calculation was performed, as sample sizes were based on prior experience with comparable experimental systems and yielded consistent, reproducible outcomes.
Data exclusions	Data were excluded only when technical artefacts (e.g., bubbles, debris, uneven illumination) interfered with image acquisition or segmentation. Exclusion criteria were defined a priori and affected ≤ 4 samples per plate. No data were excluded for biological or statistical reasons.
Replication	All key results were reproduced in at least three independent experiments performed on separate days, each including multiple technical replicates per condition. Replicate agreement was high across experiments, and all replication attempts were successful.
Randomization	Randomization was not applied because specific experimental layouts were required to assess hydrogel properties, and in drug-screening assays the order of conditions was fixed due to known drug effects. Samples were nonetheless distributed to minimize positional or edge biases within multiwell plates.
Blinding	Blinding was not needed because both experimental setup and analysis were automated and objective, minimizing operator-dependent bias.

Reporting for specific materials, systems and methods

We require information from authors about some types of materials, experimental systems and methods used in many studies. Here, indicate whether each material, system or method listed is relevant to your study. If you are not sure if a list item applies to your research, read the appropriate section before selecting a response.

Materials & experimental systems

n/a	Involved in the study
☒	☐ Antibodies
☐	☒ Eukaryotic cell lines
☒	☐ Palaeontology and archaeology
☒	☐ Animals and other organisms
☒	☐ Clinical data
☒	☐ Dual use research of concern
☒	☐ Plants

Methods

n/a	Involved in the study
☒	☐ ChIP-seq
☒	☐ Flow cytometry
☒	☐ MRI-based neuroimaging

Eukaryotic cell lines

Policy information about cell lines and Sex and Gender in Research

Cell line source(s)	Epithelial immortalized cell line (HaCaT), from Homo sapiens (Human), male, passages P50-70, provided by Professor Hugo de Jonge (Department of Molecular Medicine, University of Pavia)
Authentication	No authentication
Mycoplasma contamination	The cell line tested negative for mycoplasma contamination
Commonly misidentified lines (See ICLAC register)	/

Plants

Seed stocks	/
Novel plant genotypes	/
Authentication	/
